# Adherence to antiretroviral therapy and retention in care for adolescents living with HIV from 10 districts in Uganda

**DOI:** 10.1186/s12879-015-1265-5

**Published:** 2015-11-14

**Authors:** Nicolette Nabukeera-Barungi, Peter Elyanu, Barbara Asire, Cordelia Katureebe, Ivan Lukabwe, Eleanor Namusoke, Joshua Musinguzi, Lynn Atuyambe, Nathan Tumwesigye

**Affiliations:** Department of Paediatrics and Child Health, School of Medicine, Makerere University College of Health Sciences, P O Box 7062, Kampala, Uganda; AIDS Control Program, Ministry of Health, Kampala, Uganda; USAID SUSTAIN Project, University Research Co., LLC, Kampala, Uganda; Department of Community Health and Behavioral Sciences, School of Public Health, Makerere University College of Health Sciences, Kampala, Uganda

**Keywords:** Adolescents, HIV, Adherence, Retention, Uganda

## Abstract

**Background:**

Adolescents have gained increased attention because they are the only age group where HIV related mortality is going up. We set out to describe the level and factors associated with adherence to antiretroviral therapy (ART) as well as the 1 year retention in care among adolescents in 10 representative districts in Uganda. In addition, we explored the barriers and facilitators of adherence to ART among adolescents.

**Methods:**

The study involved 30 health facilities from 10 representative districts in Uganda. We employed both qualitative and quantitative data collection methods in convergent design. The former involved Focus group discussions with adolescents living with HIV, Key informant interviews with various stakeholders and in depth interviews with adolescents. The quantitative involved using retrospective records review to extract the last recorded adherence level from all adolescents who were active in HIV care. Factors associated with adherence were extracted from the ART cards. For the 1 year retention in care, we searched the hospital records of all adolescents in the 30 facilities who had started ART 1 year before the study to find out how many were still in care.

**Results:**

Out of 1824 adolescents who were active on ART, 90.4 % (*N* = 1588) had ≥95 % adherence recorded on their ART cards at their last clinic visit. Only location in rural health facilities was independently associated with poor adherence to ART (*P* = 0.008, OR 2.64 [1.28 5.43]). Of the 156 adolescents who started ART, 90 % (*N* = 141) were still active in care 1 year later.

Stigma, discrimination and disclosure issues were the most outstanding of all barriers to adherence. Other barriers included poverty, fatigue, side effects, pill burden, depression among others. Facilitators of adherence mainly included peer support groups, counseling, supportive health care workers, short waiting time and provision of food and transport.

**Conclusion:**

Adherence to ART was good among adolescents. Being in rural areas was associated with poor adherence to ART and 1 year retention in care was very good among adolescents who were newly started on ART. Stigma and disclosure issues continue to be the main barriers to adherence among adolescents.

## Background

Worldwide, it is estimated that about 2.1 million adolescents aged 10–19 years are living with HIV [1]. With improved access to antiretroviral therapy (ART), most of the children with vertical transmission of HIV will soon become adolescents. About 127,000 adolescents aged 10 to 19 are living with HIV in Uganda [2, 3]. Both good adherence and retention in care are a prerequisite to successful management of adolescents living with HIV (ALHIV) [4]. Poor adherence is associated with poor treatment outcome [5–7]. In the case of ART, optimal adherence is taking 95 % and above of prescribed medication [5, 8–10]. Poor adherence to ART could lead to drug resistance which translates into higher costs not only to the individual but also to the national ART programs [8, 9]. This is because when patients fail on their first line regimens, they have to be switched to more expensive second line drugs [11]. Adherence to ART among adolescents is also vital in prevention of HIV transmission [12]. Retention in care after enrollment is necessary for optimal clinical outcomes, monitoring treatment response, social support and education among others [13]. For those on ART, loss to follow up implies stopping ART with resultant disease progression, development of drug resistance and eventually increased mortality [14].

Adherence to ART among adolescents has been noted to be low [15–20]. Moreover, some studies have indicated that being well/healthy is associated with poor adherence [21–23]. In Uganda, the 2013 revised national ART treatment guidelines recommend that all those below 15 years are started on ART regardless of their immunological or clinical stage of the disease [24]. The resulting large numbers of healthy children and adolescents starting ART raises concerns about adherence to medication. In 2010, retention in care using 72 cohorts and 226,307 patients found that the 24-month retention rate in Africa was 70 % and 36-month estimate was 64.8 % [25]. This is an unacceptably low rate in resource limited settings. In Uganda, the retention in care for 617 adolescents in The AIDS Support Organization (TASO) ART programs was 96 % at 6 months and 90 % at 12 months [26]. However, these findings could not be generalized to the whole country since a lot of support including home care is provided by TASO HIV program.

In Uganda, adherence to ART is measured at each clinic visit using pill counts and recorded on a facility held HIV care/ART card that maintains a record of the client’s basic information and their follow up chronic AIDS care/ART [27]. There is therefore existing adherence data for all those on ART in their patient records at health facilities. Data on retention in care also exists in the national tools located at the health facilities [28, 29]. Adherence to ART among adolescents has been described among smaller numbers of ALHIV [17–20]. In this study, we set out to describe the level of adherence to ART and associated factors among ALHIV on a large scale involving several HIV programs representing all regions in the country. We also set out to describe the 1 year retention in care for adolescents who started ART in the third quarter of 2012. The study was strengthened by involving a qualitative aspect to help explore the barriers and facilitators of adherence to ART and retention in care among the adolescents.

## Methods

### Study design

This was a mixed methods research which employed both quantitative and qualitative methods in a convergent design. It was carried out between December 2013 and February 2014. There were two teams of research assistants sent out to the districts. Both qualitative and quantitative data collection took place at the same time in the facilities. Some research assistants collected quantitative data while others collected the qualitative data.

### Ethical statement

Written informed consent was obtained from all ALHIV aged 18 years and older, their caregivers and all respondents of KIIs. Assent was obtained from adolescents below 18 years and their caregivers consented before the adolescents participated in the study. Ethical approval was obtained from Makerere University College of Health Sciences; School of Public Health Institutional Review Board and Uganda National Council for Science and Technology before data collection.

### Study setting

This was a nationally representative study. Uganda has 112 districts distributed into 10 sub regions. All 10 sub regions of Uganda were involved in the study. These included West Nile, North, Karamoja, Eastern, East-Central, Kampala, Central 1 & 2, western and South West.

### Sampling procedure

To select 10 districts out of 112 districts in Uganda, one district was purposively selected from each sub region. These included; Arua, Gulu, Serere, Mbale, Iganga, Kampala, Kiboga, Masaka, Kiruhura and Kabarole district. Of the 10 districts, 30 % were randomly selected from districts which recorded good services (levels of Cotrimoxazole prophylaxis above 80 %, 6 months CD4 access of 80 % and linkage to care for ALHIV of 70 %). Another 30 % were selected from those without the good services and the rest were districts with unique situations according to the baseline quantitative adolescent survey by the Uganda Ministry of Health (MOH) [30].

In each district, only health facilities offering ART services for adolescents were selected. Of these, one had to be a hospital, one a Health Center (HC) IV and a HC III so that different facility levels were covered. Due to logistics, it was predetermined that we study only 30 health facilities. We purposively selected 10 hospitals of which 5 were Regional Referral Hospitals. To make 10 HC III and 10 HC IVs, we purposively selected one HC III and HC IV, from each of the 10 districts. By stratified sampling, selection of facilities was made in a way to capture those who had good services for ALHIV according to the baseline quantitative survey as earlier described, and those who do not. The selection was also made in such a way that public and private, various implementation partners were involved so that we get a better understanding of the various settings.

### Quantitative component

#### Selection criteria

For the quantitative part, all 30 health facilities were included.

#### Study variables

These included recorded adherence to ART at the last clinic visit, 1 year retention in care and factors associated with adherence to ART.

#### Data collection

Records on adherence to ART were available in 29 of the 30 health facilities. Adherence to ART was assessed in 29 health facilities using retrospective records review. The HIV care/ART cards belonging to all adolescents who were active in care (have visited the facility in the last 3 months) at the time of data collection were selected and the adherence level at the last visit was extracted. Factors associated with ART adherence like the patient’s sex, age at last visit, age at ART initiation, date of birth, date of ART initiation and ART regimen were extracted. In Uganda, at all HIV clinic visits, the attending Health care provider assesses adherence to ART by pill counts and records appropriately whether “GOOD”, “FAIR” or “POOR”adherence on the HIV care/ART card. Good adherence refers to ≥95 % adherence, fair refers to 80–95 % while poor adherence is less than 80 % [21]. The recorded adherence level at the last visit was extracted and recorded on a study tool. In the large volume ART clinics, electronic records are used. In that case, adherence levels and factors were extracted from electronic records.

All 30 health facilities were assessed for retention in care. To assess retention in care, the number of ALHIV on ART still active in care after 1 year was described. One cohort which started ART in the quarter (July to September 2012) in all the 30 health facilities was traced from the health facility records to find out the loss to follow up 1 year later. This was done after eliminating those who were transferred out and those who died. Factors that could influence retention in care like CD4 counts at initiation, clinical stage at initiation, age and regimen at initiation and entry point to HIV care were also extracted from the hospital registers.

#### Data management and analysis

Quantitative data was entered into an Epi-data (version 3.1) database which was designed with appropriate controls and validation checks. Some of the adherence data from the large programs was electronic. Analysis was done using STATA 10.0 (College Station, TX, USA). Quality assurance was ensured by using a multi-disciplinary research team, pre-testing the data collection tools, training the research assistants, crosschecking all questionnaires for completeness before leaving the health facility and field supervision during data collection.

### Qualitative component

#### Sampling

Of the 10 districts involved in the study, ALHIV were selected from 5 purposively selected districts. These were selected to cater for urban and rural adolescents, various Implementing Partners and providers whether government or private. The 5 selected districts included; Kampala, Kiboga, Gulu, Mbale and Kiruhura districts. The 69 respondents of KIIs and 40 respondents of the IDIs involved all the 10 districts.

#### Selection criteria

Only ALHIV who knew their HIV status were selected in order to avoid accidental disclosure of HIV status. In depth interviews (IDI) were carried out involving four categories of adolescents from each of the 10 districts; ALHIV who are lost-to-follow-up from HIV care, adolescents in long term relationships/married/cohabiting, in-school and out-of-school adolescents. Key Informant Interviews (KII)s with major stakeholders in adolescent health at the different levels. Policy level respondents included 3 ministries; Ministry of Health, Ministry of Education and Sports and Ministry of Gender, Labour and Social development. At the Local government, we interviewed District Education Officers, District Health Officers, HIV focal persons and politicians. Some development partners, United Nations Agencies and donors and Implementing partners were also interviewed. Relevant community members from schools (school teachers, Head teachers and school nurses), religious leaders, local politicians, village health teams and relevant Civil Society Organizations were interviewed. At facilities, a variety of health workers involved in HIV care, counselors and heads of hospitals and other health facilities were interviewed.

#### Data collection

We conducted 33 Focus Group Discussions (FGD) involving 227 ALHIV and 5 FGDs with 46 caregivers of ALHIV. Adolescent FGDs were held in homogeneously constituted categories according to sex and age in order to allow exploration of age and gender specific needs, perceptions and experiences. Two age group categories were used; young adolescents aged 10 to 14 years and older ones aged 15 to 19 years. We also included one FGD from each of the 5 selected districts for caregivers of ALHIV. FGD data collection took place at the health facilities. All the discussions were guided by a moderator who used a FGD guide, and had an observer who took additional notes. Anonymity was maintained and confidentiality was ensured and discussions took place in the appropriate local languages.

We conducted 69 KII and 40 IDIs. The ALHIV who were lost to follow up were identified from facility records and then traced with the help of community volunteers working with the facilities. Both the KIIs and IDIs helped identify barriers to adherence among different categories of ALHIV. All FGD, IDI and KII sessions were audio taped and transcribed.

#### Data management and analysis

For the qualitative part, transcription of all the recordings was done. After that, all transcripts were translated into to English. Analysis of transcripts was done using Computer based analysis; Atlas-ti software. We used a team approach to analyse this data. First, an analysis plan was developed based on study objectives. Then we generated a coding scheme after reading a few transcripts. Our team agreed on the code definitions to avoid double meanings. This approach enhanced coding consistency. We created a project in Atlas.ti. We used the developed codebook but allowed open coding for emerging codes which were agreed and hermeneutic units developed. The codes were independently examined by an independent reviewer who was not part of the team. We run query reports for each theme and used them in writing the results. Primary documents matrices were also produced and patterns in the data observed.

Quality assurance was ensured by using a multi-disciplinary research team, training of all research assistants before data collection and field supervision during data collection. For the qualitative part, all FGDs, KIIs and IDIs were audio recorded to allow for accurate transcription. Furthermore, two independent raters analyzed the data in order to increase reliability. In addition, all the FGD guides and the IDI guides were translated to the appropriate local language.

## Results

### Quantitative results

Altogether, there were 1824 ALHIV who were active in HIV care in the 29 health facilities. One facility did not have data on adherence to ART. Table 1 shows the baseline characteristics of the ALHIV who were included in the study. Majority of those receiving ART were girls (62.6 %) and almost half were receiving care from RRHs and majority (62.7 %) were from government facilities. Most adolescents started ART late at 11 years of age on average and majority had been taking ART for only 2 years.

**Table 1:** Characteristics of ALHIV active on ART in the Health facilities

Variable		Frequency	Percent
Sex	Female	1142	63 %
	Male	682	37 %
		1824	100 %
Level of health facility	Health Centre III	24	1 %
	Health Center IV	187	10 %
	HOSPITAL	60	3 %
	RRH^a^	888	49 %
	Special Clinic	665	37 %
		1824	100 %
Health facility ownership	Government	1144	63 %
	Private	680	37 %
		1824	100 %

^a^ is a regional referral hospital

### Level of adherence to ART

**Fig. 1:**
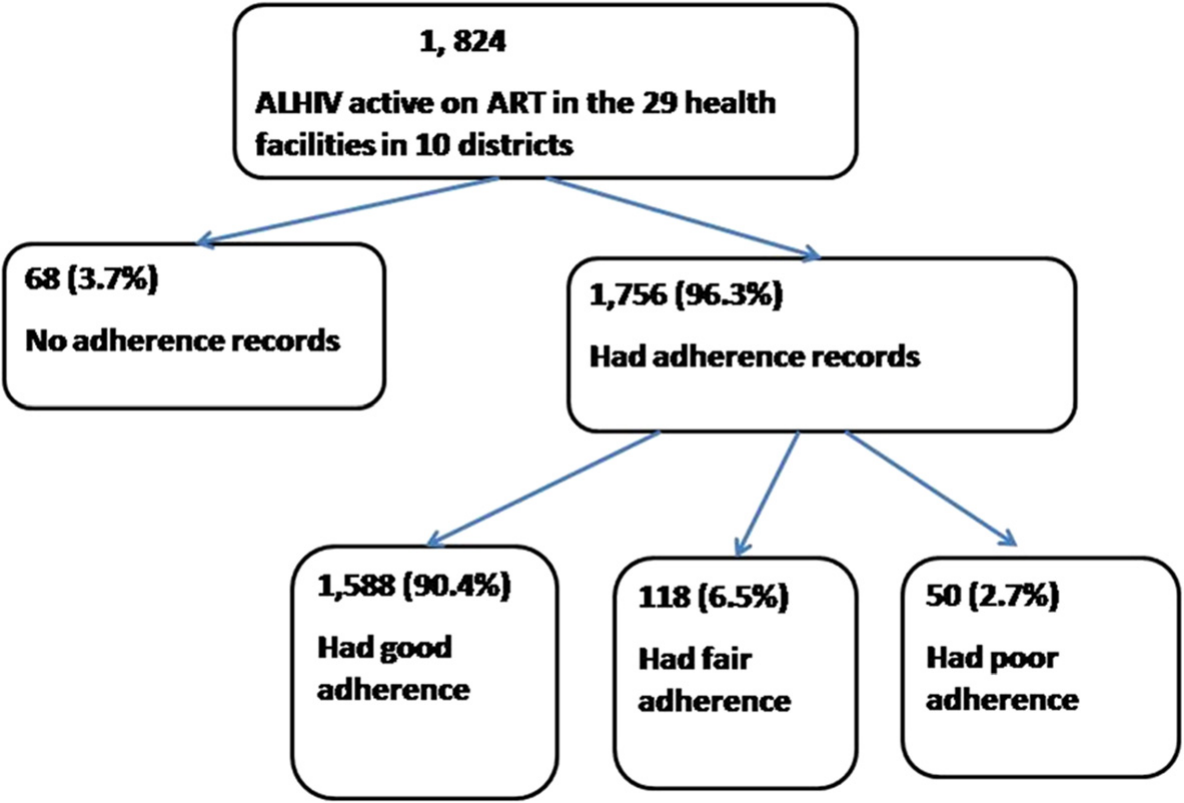
Flow diagram showing adherence to ART among the adolescents involved in the study

Adherence to ART was generally good in this study as summarized in Fig. 1. Overall, of the 1824 ALHIV, 87.1 % (*N* = 1588) had good adherence recorded on their ART cards at the last clinic visit. However, on exclusion of the 68 who had no adherence records, 90.4 % had good adherence recorded on their ART cards. We found that 6.5 % (*N* = 118) recorded fair adherence and 2.7 % (*N* = 50) recorded poor adherence at the last clinic visit. Missing records were only 68 (3.7 %).

### Factors associated with adherence to ART

Factors significantly associated with poor adherence to ART on bivariate analysis included male sex, private health facilities, health centers, rural health facilities, less than 1 year on ART and under 3 years on ART. The ART regimen at initiation did not affect adherence as shown in Table 2 below. However, on multivariate analysis, only location of facility remained statistically significant, with rural health facilities being associated with poor adherence (adjusted OR 2.64 [1.28 5.43], *P* value = 0.008).

**Table 2:** Factors associated with Non-adherence to ART in bivariate and multivariate analysis

Variable		Non adherence *N* = 168 (9.6 %)	Adherence *N* = 1588 (90.4 %)	Totals *N* = 1756	Crude OR (95 % CI)	*P* value	Adjusted OR (95 % CI)	*P* value
Sex	Female	93 (8.5)	1003 (91.5)	1096				
	Male	75 (11.4)	585 (88.6)	660	1.38 [1.00 1.90]	0.048	1.33 [0.84 2.13]	0.224
Facility location	Urban	132 (8.4)	1441 (91.6)	1573				
	Rural	36 (19.7)	147 (80.3)	183	2.67 [1.78 4.01]	0.000	2.64 [1.28 5.43]	**0.008**
Age at initiation	<15 years	122 (9.1)	1224 (90.9)	1346				
	≥15 years	46 (11.2)	364 (88.8)	410	1.27 [0.88 1.81]	0.195		
Age at last encounter	<15 years	89 (10.4)	765 (89.6)	854				
	≥15 years	79 (8.8)	823 (91.2)	902	0.82 [0.60 1.13]	0.237		
Ownership of Health Facility	Government	88 (7.9)	1022 (92.1)	1110				
	Private	80 (12.4)	566 (87.6)	646	1.64 [1.19 2.25]	0.002	0.55 [0.06 4.77]	0.585
Level of HF	Hospital	52 (5.6)	874 (94.4)	926				
	Health Center	37 (18.6)	162 (81.4)	199	3.83 [2.43 6.04]	0.000	2.02 [0.97 4.22]	0.061
Duration on ART	>1 year	85 (8.2)	949 (91.8)	1034				
	≤1 year	83 (11.5)	639 (88.5)	725	1.45 [1.05 1.99]	0.022	1.56 [0.81 3.02]	0.183
	>3 years	42 (7.0)	559 (93.0)	601				
	≤3 years	126 (10.9)	1029 (89.1)	1155	1.63 [1.13 2.34]	0.009	0.63 [0.31 1.27]	0.195
Regimen at initiation	EFV based	82 (10.3)	718 (89.7)	800				
	NVP based	82 (9.1)	821 (90.9)	903	0.87 [0.63 1.20]	0.415		
	LPV/r based	4 (11.1)	32 (88.9)	36	1.09 [0.37 3.17]	0.868		

*HF* health facility, *EFV* Efavirenz, *LPV*/*r* Lopinavir/ritotanir, *NVP* Nevirapine

The value in bold is the only one which is statistically significant and was intentionally put in bold in order for it to stand out

### Retention in care

Adolescents were generally testing and enrolling in HIV care late. Table 3 below shows that adolescents generally enroll in care at 12 years and start ART at a median age of 14 years. The girls are slightly older than the boys at testing, enrolment and starting ART. Adolescents also start ART at a low mean CD4 of 278 cells.

**Table 3:** Characteristics of Adolescents who started ART in the 3rd Quarter of 2012

Variable		Mean	Median (IQR)
Age at testing for HIV	Female	13	14 (10 17)
	Male	11	11 (9 14)
	All	12	12 (10 16)
Age at enrolment in HIV care	Female	13	14 (10 17)
	Male	11	11 (9 14)
	All	12	12 (10 15)
Age at ART initiation	Female	14	15 (12 17)
	Male	13	13 (11 15)
	All	14	14 (11 17)
CD4 at ART initiation	All	278	277 (164 348)
Variable		Frequency	Percent
Sex	Female	105	67 %
	Male	51	33 %
	Total	156	100 %
Age groups	10 to 14	81	52 %
	15 to 19	75	48 %
	Total	156	100 %
WHO staging at ART Initiation	I	15	9 %
	II	84	54 %
	III	37	24 %
	IV	14	9 %
	Missing	6	4 %
	Total	156	100 %
Level of health facility	Health Center III	1	1 %
	Health Center IV	24	15 %
	HOSPITAL	9	6 %
	RRH	49	31 %
	SPECIAL CLINIC	73	47 %
	Total	156	100 %
Health facility ownership	Government	82	53 %
	Private	74	47 %
	Total	156	100 %

*RRH* Regional Referral Hospital

Retention in care after 1 year was good in this study as shown in Table 4. Of the 156 who adolescents were started on ART in the third quarter of 2012, only 1 % (*N* = 2) died and 5 % (*N* = 7) were lost to follow up 1 year later. However, the “loss to follow up” numbers were too low to have meaningful statistical analysis. Majority of those who started (54 %) had World health Organization (WHO) clinical stage II. The table also shows that there were many more girls who started ART than boys at a ratio of 2:1.

**Table 4:** One year Outcome of the adolescents who started ART in 3rd Quarter of 2012

Outcome	Frequency	Percent
Alive	141	90 %
Dead	2	1 %
LTFU^a^	7	5 %
Stopped	0	0 %
Transferred out	6	4 %
Total	156	100 %

^a^Loss to follow up

### Qualitative results

#### Barriers to adherence to ART

Adherence challenges reported in the order of importance by ALHIV and their caretakers included stigma, laxity, poverty, side effects, disclosure challenges, pill burden in that order were the main challenges to ART adherence. On the other hand, service providers and Implementing Partners reported disclosure challenges as the main barrier to adherence.

##### Stigma

Further qualitative analysis shows that most respondents reported stigma as one of the biggest challenges to adherence to ART. Stigma is a hindrance to disclosure because ALHIV fear rejection by their friends, ridicule and discrimination. They experience statements like; “*You are positive, you have no use in future*” [**IDI**-**Gulu**-**ALHIV**-**Female**]. Others said they felt stigmatized by neighbors who whenever they would see them going to the health facility they say, “*…..there goes the HIV positive one going to get ARV drugs*”. **FGD females ALHIV Gulu**. Because of stigma, some ALHIV fear to take ARVs and may avoid the HIV clinic out of fear of meeting familiar people who would spread the news.

##### Barriers to adherence at home

The situation at home may be a hindrance to adherence. For instance, some of those staying with non biological parents as caregivers expressed mistreatment and abuse from them. Some of these caregivers are insensitive and they publicly disclose the HIV status of ALHIV. Others expressed being discriminated by their HIV negative siblings. One female ALHIV said, “*… they isolate us by refusing to share razor blades or safety pins with us. They do not want to sleep with us or even eat food with us. They fear to touch my plate and keep it separate from others (plates)*”. **IDI female ALHIV Kiboga** Lack of privacy in homes also hinders adherence to ART. In addition, older caregivers like grandparents forget clinic appointment dates.

##### Disclosure issues

The fear of rejection by their partner is a big hindrance to disclosure. Some boys said that they would rather have pleasure with girls than receive ART with a risk of their HIV status being discovered. Non disclosure to partners is a hindrance to adherence and retention in care. One adolescent girl said, “*For me, it has ever happened to me. When I was living with my boyfriend, I used to take my medicine in hiding because I never wanted him to know that I had HIV. When the medicine got finished, I feared to go to the hospital to get more. That is how I stopped taking medicine (ARVS) for about two months.*” **FGD ALHIV**-**Gulu**. Delayed disclosure of HIV status by parents or guardians also leads to poor adherence. One caregiver feared to tell the child because she thought he could easily hurt himself.

##### Barriers arising from characteristics of adolescent stage

Self image, identity and peer pressure were mentioned as barriers to adherence. One caregiver said that identity and self image affect adolescents and can therefore be a barrier to adherence. He stated, “*For example if one makes a comment to an adolesccent that, “You used to have good skin, how come you are now dark? You might be sick”. This could lead them to abandon the medicine; perceiving them as the cause of their skin and body changes. So they leave them.*” **FGD caregivers Kiruhura**. Others said peer pressure could lead an adolescent to take drugs of abuse like marijuana and go for dances which could stop them from taking their ARVs.

##### Depression and frustration

Caregivers also reported that some adolescents feel that they are as good as dead and are therefore not motivated to take the medicine. “*Some youths rationalize that since the medicine does not remove the virus from their bodies, they either leave it completely or they swallow haphazardly. They do not appreciate the benefits of taking the medicine. They take it when they remember; but if they forget and don’t take it, no problem*”. **FGD caregivers Kiruhura**.

##### Barriers at school or work

Adolescents reported stigma and discrimination at school by teachers and fellow students. Those in boarding schools lacked privacy to take their medication, which leads to missing doses. For those who do not disclose their HIV status to the school authorities, it is very difficult to get permission to leave school to keep a clinic appointment. This could lead to absenteeism and eventually drop out from HIV care. For the working adolescents, they do not have job security and they perceive that people label them as weak and do not want to employ them.

##### Fatigue

Some ALHIV start ART when they are weak and at that point they have good adherence. As they improve, their commitment to taking the medication reduces. One hospital administrator said that laxity was inevitable among adolescents since they are still young and short sighted about their future. Others said that because some ALHIV look very good, they do not seem to appreciate the need to keep taking medication. Adolescents reported that by nature they are very playful and easily forget their medication. For instance, some prefer to go to games instead of going to receive treatment. Adolescent girls said that some boys stubbornly refuse to take the medicine as stated, “*…if it meant death let them die*”. **FGD ALHIV**-**females Kampala**. They reported that some adolescents were proud and felt they did not need to swallow drugs. Others simply stopped taking the medication out of fatigue especially among those who started when they were still young as suggested by an official from the Ministry of Health.

##### Poverty

All the focus groups and key informants mentioned poverty as a key factor in ART adherence. Because of poverty, some of them lacked food and they report abdominal pain whenever they take the medicine on an empty stomach. Coupled with lack of transport to the facilities especially in the rural areas, this leads to loss to follow up or poor adherence to treatment.

##### Barriers due to the ARVs

Adolescents reported that side effects, drug palatability and pill burden are a hindrance to adherence. Others said that some of the medicines have a terrible smell and they could not take them. They feel like the medicine is “torturing” them. During FGDs with ALHIV, they indicated that taking the pills every day and on time was very difficult since ARVs do not result in a cure. The many tablets, the unpalatable taste, the size and colour caused a lot of distress. One male ALHIV said, “*There are some ARVs that are big. They may tell you to swallow 2 in the morning, 2 in the evening. …How can I swallow these drugs for the rest of my life? Moreover I am not going to change my HIV status. That is why some people stop taking ARVs.*” **IDI ALHIV**-**male**-**in**-**school Mbale**.

##### Lack of psychosocial support

This was mostly mentioned by health care providers, and other key informants. They indicated that there was no adequate psychosocial support to meet the needs of teenagers about adherence especially for those who attend boarding schools and take ARVs.

##### Myths and misconceptions

Few focus groups and key informants mentioned this as a barrier to adherence. Some thought that if they took ARVs for some time the virus would die and they would live a normal life. Others doubted their HIV results and were not eager to take the drugs. Others believed in faith healing and stopped taking medication. Some adolescents have misconceptions that ARVs kill or are so strong that they make them sicker. One village leader said there was a perception on their village that whoever tested positive would be given medication to kill them very quickly and this would discourage them from taking ARVs.

#### Facilitators of adherence and retention in care

Adolescents and their caregivers reported that peer support groups are very supportive of adherence. Presence of counselors and caring health workers also helps them to adhere to their medication. Other facilitators included scheduling clinic visits during school holidays, providing food support, transport to clinics, short waiting time, telephone calls from the facilities and text messages. Some programs motivate adolescents by teaching them skills and engaging them in income generating activities. One urban program engages adolescents in conversations with other ALHIV in other countries through social media like *google hang out* and “facebook”.

## Discussion

On exclusion of the 68 who had no records on adherence level, 90.4 % of those who had an adherence record had good adherence. This level of adherence was higher than expected for adolescents because when compared with younger children and adults, HIV-infected adolescents and young adults consistently have disproportionately higher rates of poor drug adherence and virological failure [12, 31]. Most studies have found the 95 % adherence levels by pill counts among adolescents to range between 65 and 80 % [12–14, 16]. It may have been higher in our study because of the method used to assess adherence whereby we used records. This assessment is made by clinicians during their routine clinical practice and not study settings. To note, most HIV clinics are heavy and health workers may not be in position to accurately assess adherence by counting all pills balances from all patients. In addition, studies show that pill counts method of adherence assessment can be manipulated and adherence has been found to vary with the method of assessment used [16, 17]. A similar study among adolescents receiving ART at the Joint Clinical Research Centre in Kampala, Uganda, found that 93, 67 and 23 % of patients had an adherence of greater than 95 % as measured by self-report, clinic based pill counts and electronic cap methods, respectively [16]. This study had much lower adherence levels by pill counts than our study. In addition, about two thirds of the adolescents were on ART for less than 3 years. Studies show that adherence to ART gets worse with time [32].

Adherence was worse among the rural compared to urban living adolescents. This was contrary to findings in a cohort study from 2006 to 2011 which was conducted among 1000 children resident in urban and rural settings of Uganda to compare the response to ART among urban versus rural children and the factors associated with this response. Adherence of ≥95 % was observed in 88.2 % of urban versus 91.3 % of rural children by self-report, and in 78.8 % of urban versus 88.8 % of rural children by pill counts. This study also reported that rural children had more favorable clinical outcomes and were more likely to adhere optimally to ART than urban children [33]. However, our study consisted of adolescents who are a special group that needs a lot of psychosocial support. The urban located facilities in our study had more adolescent friendly services, peer support groups and more innovations to support ALHIV than in the rural facilities which could explain the better adherence. Studies show that adolescents are attracted to such adolescent friendly services [34, 35].

One year retention in care was very good in this study with only 5 % lost to follow up and 1 % mortality. We could not analyze the factors associated with loss to follow up since the numbers were too few. The retention in care could have been very good because almost 80 % of the ALHIV were in special clinics and RRHs which have a lot of support from partners in terms of making services adolescent friendly, telephone follow up, providing food and even transport in some programs. Studies have shown that support given to clients improves retention in care [14]. Retention in care was much higher than what studies in Africa have shown [19]. However, it was similar to what was found in TASO ART program where retention was 96 % at 6 months, 90 % at 12 months, 83 % at 24 months, 76 % at 36 months, and 71 % at 48 months [20]. The study period of 1 year could be responsible for this finding and retention would probably reduce with time. Furthermore, studies show that being on ART is associated with better retention in care. [36]. In our study, we only involved ALHIV who were taking ART. Our mortality rate was very low, contrary to what studies show that the mortality amongst ALHIV is high [37]. For instance, one observational prospective cohort study of adolescents and adults with HIV (>15 years) at a community-based ART clinic in South Africa reported the probability of death in the first year of ART (7.9, 95 % CI 7.0–8.9 %) [38]. This difference could be because 63 % of ALHIV started ART with WHO clinical stage 1 and 2 and 68 % were attending care at specialized HIV clinics and Regional referral clinics, coupled with the high levels of ART adherence from our study.

From the qualitative component of the study, several barriers to adherence were reported in the homes, school and health facility. Among them, disclosure, stigma and discrimination were the most predominant barriers. Disclosure has been described to be a facilitator of adherence among children [39]. Similar to other studies, the challenges to ART adherence among ALHIV were multifactorial and included family situations, socioeconomic factors, medication issues and healthcare systems [40–43].

The strength of this study is that it had both qualitative and quantitative aspects which yielded very helpful data. It involved the ALHIV, their caregivers, schools and others in the education sector, various health workers and resource persons. It also had good representation since all 10 sub regions of the country were involved in the study. Furthermore, rural and urban as well as public and private facilities were included in the assessment. However, we encountered a few limitations. First, we used the recorded adherence level from the clinical records which may not be as accurate as measuring adherence in a study setting. However, since these records are available and adherence is assessed at every visit, it was important to use them to describe the adherence levels from a large sample of ALHIV. We also found gaps in the data for the retrospective records review. Lastly, information was gathered from only 10 out of 112 districts in the country due to limited resources. However, efforts were made to ensure regional representation by involving one district per region.

## Conclusion

Adherence to ART among adolescents was very good with over 90 % recording good adherence. Adolescents residing in rural areas have less support and were associated with poor adherence. Stigma and discrimination were the main barriers to adherence amongst adolescents. One year retention in care was very good with 90 % of ALHIV still alive and in care 1 year later. We recommend another study to describe the retention in care for adolescents after longer durations on ART.

## Abbreviations

AIDS: Acquired Immunodeficiency Syndrome
ALHIV: Adolescents living with HIV
ART: Antiretroviral therapy
FGD: Focus Group Discussions
HC: Health Center
HIV: Human Immunodeficiency Virus
IDI: In depth interviews
KII: Key Informant Interviews
MOH: Ministry of Health
SUSTAIN: Strengthening Uganda’s Systems for Treating AIDS Nationally
TASO: The AIDS Support Organization
USAID: United States Agency for International Development
WHO: World Health Organization
